# Efficacy of a children’s procedural preparation and distraction device on healing in acute burn wound care procedures: study protocol for a randomized controlled trial

**DOI:** 10.1186/1745-6215-13-238

**Published:** 2012-12-12

**Authors:** Nadia J Brown, Sylvia Rodger, Robert S Ware, Roy M Kimble, Leila Cuttle

**Affiliations:** 1 Centre for Children’s Burns and Trauma Research, Queensland Children’s Medical Research Institute, University of Queensland, Royal Children’s Hospital, Brisbane, Queensland, Australia; 2 Division of Occupational Therapy, The University of Queensland, School of Health & Rehabilitation Sciences, Brisbane, Queensland, Australia; 3 The University of Queensland, Queensland Children’s Medical Research Institute, Brisbane, Queensland, Australia; 4 The University of Queensland, School of Population Health, Brisbane, Queensland, Australia

**Keywords:** Burns, Child, Pain, Stress, Anxiety, Salivary cortisol, Salivary alpha-amylase, Virtual reality, Randomized clinical trial

## Abstract

**Background:**

The intense pain and anxiety triggered by burns and their associated wound care procedures are well established in the literature. Non-pharmacological intervention is a critical component of total pain management protocols and is used as an adjunct to pharmacological analgesia. An example is virtual reality, which has been used effectively to dampen pain intensity and unpleasantness. Possible links or causal relationships between pain/anxiety/stress and burn wound healing have previously not been investigated. The purpose of this study is to investigate these relationships, specifically by determining if a newly developed multi-modal procedural preparation and distraction device (Ditto™) used during acute burn wound care procedures will reduce the pain and anxiety of a child and increase the rate of re-epithelialization.

**Methods/design:**

Children (4 to 12 years) with acute burn injuries presenting for their first dressing change will be randomly assigned to either the (1) Control group (standard distraction) or (2) Ditto™ intervention group (receiving Ditto™, procedural preparation and Ditto™ distraction). It is intended that a minimum of 29 participants will be recruited for each treatment group. Repeated measures of pain intensity, anxiety, stress and healing will be taken at every dressing change until complete wound re-epithelialization. Further data collection will aid in determining patient satisfaction and cost effectiveness of the Ditto™ intervention, as well as its effect on speed of wound re-epithelialization.

**Discussion:**

Results of this study will provide data on whether the disease process can be altered by reducing stress, pain and anxiety in the context of acute burn wounds.

**Trial registration:**

ACTRN12611000913976

## Background

### Burn pain

Pain is multidimensional and highly complex, and involves the integration of sensation and perception. Emotions, individual attributes, cognitive, environmental and cultural factors, together with the child’s focus of attention and level of control, all play a significant role in diminishing or magnifying the perception of pain [1,2]. Despite considerable advances in burn wound management, procedural pain is both the most intense pain, and the most common type of burn pain to be undertreated [3]. Lack of well-established evidence-based protocols of burn pain management; inaccurate fears of addiction; infrequent pain assessment; and poor correlations between the nurse’s and the patient’s perception of pain, attribute to the occurrence of under medication in children [4]. It is not surprising that the entire wound healing period can provoke a high level of stress and anxiety, particularly for children.

### Procedural anxiety

Anxiety, which commonly co-exists with pain, impedes our coping mechanisms and ability to tolerate pain. Anxiety is a future-orientated emotion of apprehension, nervousness, tension, fear and worry, accompanying physical sensations and influencing subjective perception [5]. Highly anxious burn patients are, therefore, more susceptible to lower pain tolerance [6,7]. Several studies refer to the reciprocal relationship between pain and anxiety [8,9]. In addition to anxiety, burn injuries, which are a type of trauma, may induce acute stress symptoms, [10] and psychological disorders, including post-traumatic stress disorder (PTSD) [11].

### The effect of stress on the body

Pain and anxiety places the body under both physical and physiological stress. Stress has been reported to interrupt and delay the cascade of healing in several studies, including skin barrier recovery after tape stripping [12,13]; punch wound biopsies [14-16]; suction induced blisters [17,18] and pre-surgical stress [19]. The biological mechanisms behind this may be explained through the stress induced elevation of glucocorticoids and adrenaline and noradrenaline levels. Elevation of these stress hormones produces an immunosuppressive effect, reducing the infiltration and activation of neutrophils and macrophages [20], and also suppressing the production of proinflammatory cytokines IL-1β and TNF-α [21]. Proinflammatory cytokines are crucial to the recruitment of phagocytic cells to clear away contaminating debris; in activating and recruiting cells involved in wound healing, including lymphocytes and other macrophages; and regulating fibroblast chemotaxis, proliferation, collagen synthesis and endothelial cells involved in the repair process [22]. Attenuation of the expression of these proinflammatory cytokines is likely to impair healing through delaying the inflammatory stage of wound healing [23].

Additionally, immune function plays a pivotal role, particularly in the early processes of wound healing. Elevated glucocorticoids, adrenaline and noradrenaline alter cellular function and differentiation of T cells, causing a shift from Th1 cellular to Th2 humoral immune function [24]. Stress-induced suppression of Th1 cellular immunity may inhibit the secretion of inducible nitric oxide synthase (iNOS) in macrophages [22]. Angiogenesis, endothelial and epithelial cell proliferation and migration are central to wound healing and are highly influenced by nitric oxide [25]. Additionally, an increase in iNOS production has been shown to be just as detrimental as iNOS deficiency [26]. Stress-induced increases of adrenaline in mice were shown to heighten iNOS to cytotoxic levels, impairing wound healing [20]. A stress–induced shift to Th2 humoral immunity activates mast cells, which release histamine and result in inflammation. The Th2 profile up-regulates B lymphocytes, leading to the production of antibodies [27], rather than contributing to wound healing processes. Stress may still continue to disrupt healing after wound closure. Fibroblasts, together with their by-products collagen and matrix metalloprotinases (MMPs), comprise the main contributors to wound maturation [28]. Stressed mice displayed reduced levels of matrix MMP-2 and MMP-9, which are involved in cell migration and collagen turnover. Consequently, reduced levels may delay granulation tissue re-modeling, resulting in a less organized collagen structure (rather than being aligned parallel with the lines of contraction) and immature collagen scaffolds [20].

### Reducing pain, anxiety and stress

Dampening pain intensity, unpleasantness, anxiety and time spent thinking about pain through the use of virtual reality (VR), has created much interest since the first published case report in 1999 with adolescents during burn wound care procedures [29]. An accumulation of studies in the area of burns are reporting a discernible reduction in pain ratings when VR is used alongside pharmacological intervention [30-39]. VR encompasses multisensory stimuli from sight, sound and touch, greatly drawing the subject's attention into the virtual world, creating a sense of “presence” [31]. VR acts to psychologically dissociate the patient from pain by activating higher cognitive and emotional regions in the brain. The full extent and awareness of pain is reduced, as seen in functional magnetic resonance imaging which showed a dampened transmission to primary regions involved in emotional processing of pain (caudal anterior cingulated cortex) and the sensory component of pain (primary somatosensory cortex) [40]. Distraction has also been shown to gate pain perception through activation of the periaqueductal gray, which was not activated during pain stimulus alone [41].

The multi-modal distraction Ditto™ (Diversionary Therapy Technologies, Queensland, Australia) is a medical device that has a preparatory and distraction phase [42]. The preparatory phase involves the child engaging in the story “Bobby gets a Burn©”. This interactive story has been specifically tailored for 3- to 12-year-olds to explain the clinical procedures and sensory phases of a burn dressing change in a child-focused manner. The story aims to reduce anxiety, fear and distress through exposing and desensitizing the child to the procedure; acknowledging feelings associated with the sensory aspects of the procedure; and instilling a sense of control through equipping the child with the knowledge of what will transpire. Following the preparatory story, conducted in the waiting room, the child engages in a choice of interactive stories or games throughout wound care procedures, forming the distraction phase of the Ditto™. A large degree of the success of the Ditto™ device is due to its novel design [42], and the unique and child-friendly educational content concerning the procedure [34]. Off-the-shelf virtual reality systems have not previously been able to significantly decrease burn pain scores in adolescents [43]. However, the novel and customized content and technology of the Ditto™ has proven effective in reducing pain levels compared to off-the-shelf video games [34].

Results from Miller’s trials [33,34] confirmed the effectiveness of the Ditto™ in significantly reducing pain ratings and treatment length. Retrospective review of medical notes identified the Ditto™ treatment group wounds re-epithelialized an average of two days faster than the standard distraction group [33]. This exciting and clinically significant finding highlights the potential of this Ditto™ device with pain reduction and also possibly improving wound healing. In the Miller trial, burn wound depth was not matched between control and treatment arms and the measurement of wound re-epithelialization was only obtained retrospectively from the chart notes. The possible link between stress and burn wound healing have stimulated the development of this current trial to measure stress, wound healing and pain in a scientific and controlled manner.

### Objectives

The aim of this study is to determine whether use of the Ditto™ device is associated with the rate of burn wound healing (re-epithelialization).

## Methods

### Design

**Figure 1:**
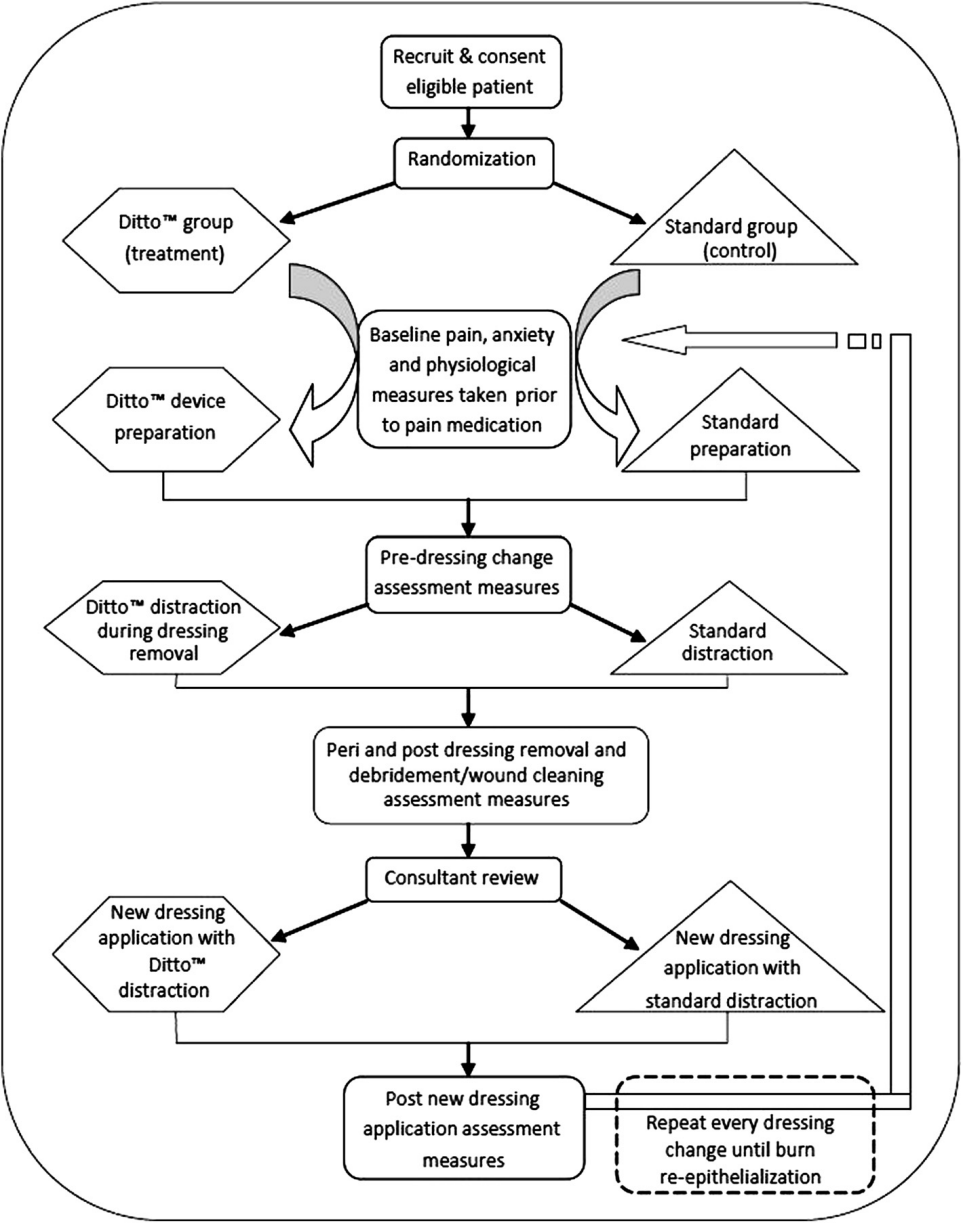
**Study design flow chart.** The sequential order and timing of data collection within the context of burn wound care procedures.

This study is a prospective, superiority, randomized controlled trial, consisting of two parallel groups. Participants will be randomized to receive either (1) standard preparation and standard distraction (control group), or (2) the Ditto™ device, including preparation and distraction phases (treatment group), to test the superiority of this new non-pharmacological intervention on acute burn wounds. The data collection design is displayed in Figure 1.

This trial protocol was ethically approved by both the Queensland Children’s Health Services (Royal Children’s Hospital) Human Research Ethics Committee and The University of Queensland Ethics Committee, and was registered with the Australian New Zealand Clinical Trials Registry (ACTRN12611000913976).

### Setting and participants

Participants are being recruited from the Stuart Pegg Paediatric Burns Centre at the Royal Children’s Hospital, Brisbane, Australia. Children presenting to this center from August 2011 will be screened on admission for eligibility to this trial.

### Inclusion criteria

Participants who are aged between 4 and 13 years with an acute burn injury of any depth and a burn total body surface area of <15%, presenting on their first dressing change, will be considered for inclusion in this study.

### Exclusion criteria

Children will be excluded from the study if they are non-English speaking; have a cognitive, visual or auditory impairment, or a diagnosis under the Autism Spectrum Disorders; have a diagnosed illness in addition to a burn injury; have been reported to the Suspected Child Abuse and Neglect (SCAN) system (as these children may have additional emotional and psychological issues affecting stress, anxiety and coping mechanisms); receive sedative medication (Midazolam, Entonox™ (BOC Healthcare, Worsley, Manchester, UK)); and if their burns require grafting. Several of these exclusions will not become known until after patient recruitment and randomization due to the nature of the clinic and the inability to predict patient and wound management needs prior to dressing removal.

All eligible children will be invited to participate. Participation in the study will not alter the standard medical treatment received.

### Interventions

Various outcome measures and saliva samples will be collected at several time points during the burn wound care procedures (Table 1), with the intervention (Ditto™) used prior to treatment as preparation and during treatment as a distraction at every change of dressing. 

**Table 1:** Schedule of measurements

**Outcome measures**	**Waiting room**	**Pre-DR**	**DR**	**Post-DR**	**Consult**	**New DA**	**Post new DA**	**3 months post healing**
Salivette®	X			X				X
FLACC	X	X		X			X	
FPS-R	X	X		X			X	
VAS-A	X	X		X			X	
Heart rate	X		X	X		X	X	
Oxygen saturation	X			X			X	
Parent demographic questionnaire	X							
CTSQ (1^st^ COD only)	X							X
LDI (1^st^ COD only)				X				
Photos				X				
Visitrak™				X				
Time taken			X		X	X		
Dressing used						X		
Parent satisfaction questionnaire							X	
Ditto™ Enjoyment Scale							X#	

The outcome measures taken at each time point over the course of the study.

X = measure taken, # = Ditto™ intervention group only. COD, change of dressing; CTSQ, Children Trauma Screening Questionnaire; DA, dressing application; DR, dressing removal; FLACC, Faces, Legs, Arms, Cry, Consolability; FPS-R, Faces Pain Scale-Revised; VAS-A, Visual Analog Scale–Anxiety; LDI, laser Doppler image.

### Waiting room

A baseline saliva sample 1 will be obtained in the waiting room prior to nursing administration of pharmacological pain relief in accordance with standard practice protocols within the Burn Centre (primarily oxycodone, an opioid derived pain medication, dosage determined by body weight, 0.1 to 0.2 mg/kg). Saliva samples will be collected with Salivettes™ (Sarstedt Australia Pty, Ltd. Mawson Lakes, SA, Australia), by placing the synthetic roll in the child’s mouth for a period of two minutes. Baseline measures will be taken in the waiting room for: heart rate (HR); oxygen saturation; pain ratings from the nurse and the child; a self-report anxiety measure from children eight years and over; height and weight. Demographic information and pertinent clinical characteristics will be obtained from the caregiver and patient chart: mode and site of injury; total body surface area (TBSA) of burn; depth of burn; burn first aid treatment received; skin color; medication administered; hours per week spent engaging in computer games and home video games. TBSA is calculated using the Lund and Browder chart [44]. Food and fluid consumption two hours prior to saliva samples will also be recorded as possible confounding variables of salivary analysis in addition to the time of sample collection and time of waking.

Participants will then be randomly allocated to one of two groups:

(a) *Treatment Group*: Ditto™ device including preparation and distraction phases.

**Figure 2:**
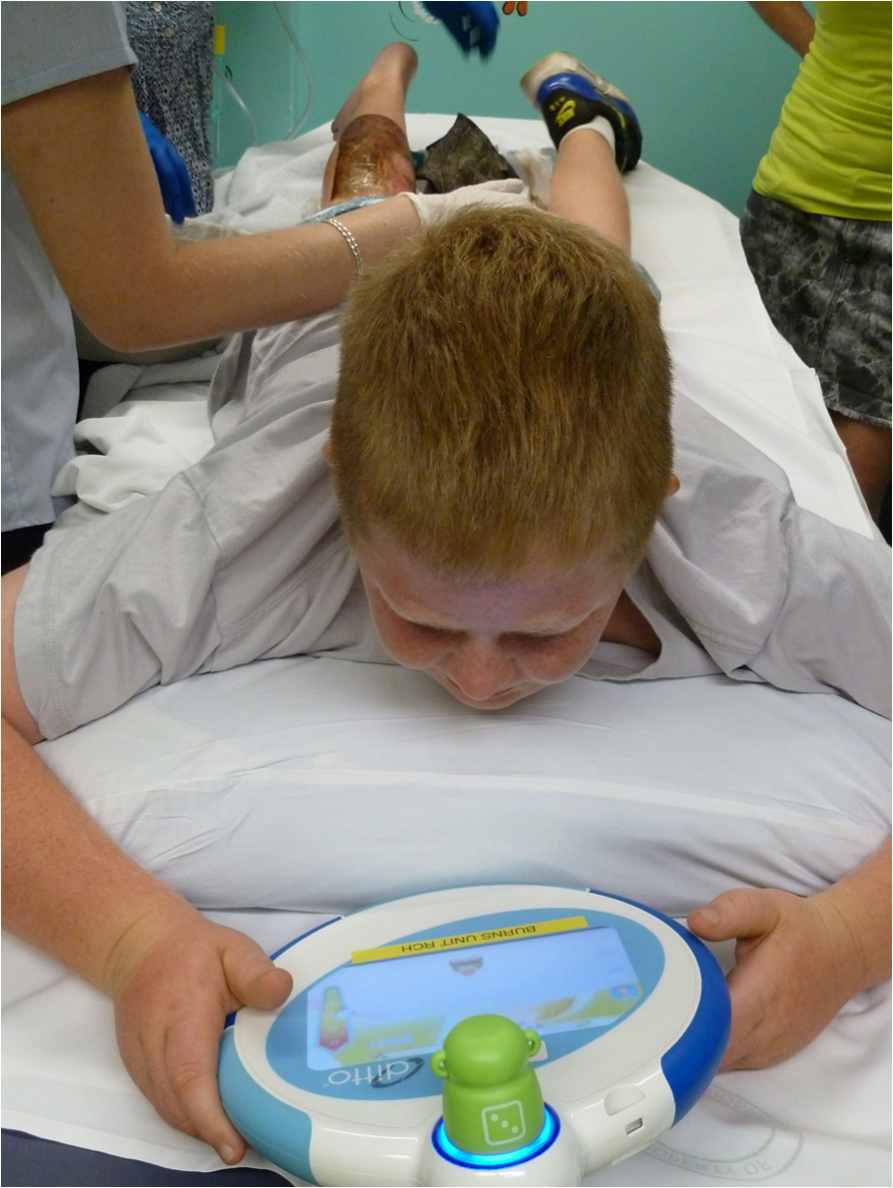
**Patient engaging in the Ditto™ distraction phase during dressing removal.** A burn patient engaging in Ditto™ distraction as nurses carry out the wound care procedures.

While waiting for medication to take effect, children will be given the Ditto™ device in the waiting area to engage in the procedural preparation story of “Bobby gets a Burn©.” Upon entering the treatment room, participants will engage in their choice of games or interactive stories on the Ditto™ device. Engagement will occur prior to the nurses commencing dressing removal procedures and continue throughout the wound care procedure. Figure 2 depicts a patient engaging in the Ditto™ device during the distraction phase of the treatment.

(b) *Control Group:* Standard preparation and distraction

The control group will have access to standard distraction, such as television, videos, books, toys and parental soothing. Nursing staff may give information before or during the process as per standard practice; however, no Ditto™ device procedural preparation or distraction will be available to the control group.

### Dressing removal

Pain, anxiety and physiological measures will be repeated prior to commencing dressing removal. All participants will have their HR recorded at two-minute intervals via an oximeter on their finger or toe. The choice of games/stories by participants in the treatment group will be recorded by the primary researcher.

### Post dressing removal

Immediately following dressing removal and debridement/wound cleaning, saliva sample 2 will be taken and, at a further 10 minutes later, saliva sample 3 will be obtained with date and time of collection documented. A retrospective measure of pain/distress and anxiety during the burn dressing removal and debridement will be taken from the child and nurse. Time taken (in minutes) for dressing removal and debridement and the number of nurses involved will also be recorded.

All participants will then have their burns scanned using a laser Doppler imager (which measures burn depth by displaying blood perfusion of tissue) on their first dressing change only. A Visitrak™ (Smith & Nephew Pty Limited, London, UK) trace of the wound area and photos will be taken at every dressing change.

### New dressing application

The type of new dressing applied will be documented and HR, oxygen saturations and the number of nurses and nursing time again recorded for the application of the new dressing. The choice of Ditto™ distraction games and stories that the treatment group engaged in will be recorded.

### Post new dressing application

Immediately following the new dressing application, nursing retrospective pain/distress ratings and child retrospective pain and anxiety ratings will be taken. Measures will continue to be taken during every dressing change (usually every three or seven days), using the same protocol as above until complete re-epithelialization. Parents will be asked to rate their level of satisfaction with pain management at the end of every dressing change with the use of a visual analog scale from “not satisfied” to “very satisfied.” Children in the Ditto™ treatment group will be asked to rate their level of enjoyment in using the Ditto™ on a 10 cm line visual analog scale. Any relevant comments made by the participant or parent/carer will be recorded.

Within the first week the Child Trauma Screening Questionnaire (CTSQ) will be completed with children six years and over through interview by the researcher. The participants will then be reviewed by a consultant as required.

### Follow-up

At the three-month follow-up, the CTSQ will be re-conducted via mail/phone, and a Salivette™ will be mailed to participants to obtain sample 4, a true baseline saliva sample (at a similar time of day to that of the pre-medication saliva sample 1) and returned via post.

### Outcomes

This study will assess the impact of the Ditto™ device on wound healing of acute burn injuries. Healing will be measured by the number of days until complete re-epithelialization, with data collection commencing on the first dressing change. Secondary outcomes will be measuring the impact of the Ditto™ device on pain, anxiety and stress.

### Primary outcome measures

#### Wound healing

The amount of wound re-epithelialization and the number of days from the date of the burn injury until when the complete wound re-epithelialization occurs will be measured by (a) the consultant’s clinical judgment; (b) blinded review of photographs; and (c) the Visitrak™ (Smith & Nephew) grids. Photos will be taken at every dressing change with inclusion of a ruler and a grey scale (QPcard 101 v2, Kayell, VIC, Australia). Photograph lighting levels will be standardized using Adobe® Photoshop® Elements 9 (San Jose, CA, USA)) to enable accurate comparison of wound colors across photographs. Blinded review of photos by a panel of burn wound specialists to assess re-epithelialization and general wound appearance will occur upon cessation of data collection. The Visitrak™ grids will be used to trace around the wet (un-re-epithelialized) and dry (re-epithelialized) areas of the wound.

A laser Doppler scan will be performed on the first dressing change only, following debridement, to accurately measure wound depth, allowing for comparison of wounds across participants. Burns covering more than one body part will be scanned separately. In addition, more than one scan will be performed when burns are circumferential or extend around curved surfaces in order to capture accurate frontal, medial and/or lateral views as appropriate. The MoorLDI2-BI2 laser Doppler imager (LDI), Moor Instruments Limited, Devon, UK, contains a visible red laser diode target beam of wavelength 660 nm, and a near infra-red laser diode for measurements by the laser Doppler with a wavelength of 780 nm. All scans will be performed in burn treatment rooms maintained at a range of 22 to 24°C. A dark green sterile surgical drape will be placed as a background underneath the area to be scanned. The LDI scanner head will be positioned approximately 35° off perpendicular at a scanning distance range of between 40 and 70 cm from the wound and set on the fast scan resolution setting. The onboard software package (moorBDA v2.4, Moor Instruments, Axminster, Devon, UK) will be used to calculate different wound depths (perfusion units) as a percentage of total wound area.

### Secondary outcome measures

#### Pain

Pain will be assessed before, during and after wound care procedures by obtaining the participant’s self-report of pain intensity using the Faces Pain Scale – Revised (FPS-R); the nurse’s behavioral/observational rating using the Face, Legs, Arms, Cry, Consolability (FLACC) scale; and physiological indicators, including heart rate and oxygen saturations. The Faces Pain Scale – Revised (FPS-R) was chosen for this study over other pain scales (for example, the Wong-Baker FACES Pain Scale) due to its high clinical utility and psychometrically sound properties [45].

Behavioral measures are an important tool to use as an adjunct to self-report scales, particularly in children who may be sedated by drugs; have a cognitive or communication impairment; or are too young to comprehend a self-report scale. In addition, a child’s self-report may be exaggerated, diminished or altered due to cognitive, emotional or environmental and situational factors [46]. The Face, Legs, Arms, Cry, Consolability (FLACC) scale was chosen as it shows excellent responsiveness (detecting significant change in pain scores), reliability, content and construct validity [46].

#### Anxiety and fear

Emotional responses encompass negative affect and emotional facets secondary to pain, including anxiety, distress and fear. The fear thermometer [47] is an anxiety measure that may be used with younger children; however, it was rejected as a measure as children under eight years are reported to have difficulty cognitively distinguishing between the sensory experience of pain (pain intensity) and the affective response (distress, anxiety, fear) to pain [48]. An anxiety measure will only be taken from children eight years and above, using the Visual Analog Scale-Anxiety (VAS-A). The VAS-A has been validated as an accurate self-report of anxiety for burn injuries [49], as well as other patient populations [50-53], and is more sensitive to change over time for pediatric studies [54,55].

#### Stress

Salivary cortisol (reflecting the hypothalamic-pituitary-adrenal axis activity) and salivary α-amylase (reflecting sympathetic nervous system activity) will be used as biological markers of stress levels during burn wound care procedures. Salivettes® without citric acid (Sarstedt Australia Pty. Ltd.) will be used to collect saliva at three time points: baseline in the waiting room prior to administration of pain medication; at 0 minutes following dressing removal and debridement to capture the peak salivary α-amylase levels; and at 10 minutes to capture the peak cortisol HPA axis activity. These time points were identified in a pilot study of 10 patients. At these time points the absorbent synthetic roll will be placed in the child’s mouth for a period of two minutes. Date and time of collection will be recorded and samples will be refrigerated at 2°C and processed within seven days. Samples will be spun in a centrifuge at 3,000 rpm at room temperature (22°C) for 10 minutes and the saliva frozen at -80°C until analysis by Queensland Pathology. Ultra high performance liquid chromatography-tandem mass spectrometry will be used to analyze salivary cortisol [56] and Amylase EPS-G7 Reagent (Thermo Scientific, Middletown, VA, USA) used to measure salivary α-amylase, performed according to the manufacturer’s instructions.

Detection of PTSD in children remains challenging as the DSM-IV criteria for PTSD has been defined and tested on adults [57] and shows lack of sensitivity in diagnosing posttraumatic stress symptoms in young children [10]. Screening tools were the only feasible measure for this study due to clinical utility and the time constraints of the setting. The Child Trauma Screening Questionnaire (CTSQ) [58] is a self-report tool for children and adolescents 6 to 16 years, based on the 10-item Trauma Screening Questionnaire for adults [59]. The CTSQ screens for hyper-arousal symptoms and for re-experiencing symptoms following the traumatic event [60]. The CTSQ is more accurate than the Children’s Impact of Events Scale – version 8 in predicting PTSD at one month and six months after injury and diagnosing full and sub-syndromal PTSD [58].

#### Treatment satisfaction

Engagement, interaction and appropriate use of the Ditto™ will be measured by participant satisfaction. If a participant refuses to use the Ditto™ they will be excluded from the study. If, however, a child accepts the use of the Ditto™ and appears disinterested and does not completely engage with the Ditto™, this will be reflected in the child’s rating of how much they liked using the Ditto™. Other measurements of engagement were considered such as video recording, motion monitors measuring limb activation, and frequency measures of time spent looking away from the device. These latter measures were unsuitable for the Burn Centre outpatient environment and contradicted the specific design of the Ditto™ [42], which enables the child to interact with their caregiver, receive reassurance or check on the wound care procedures at any stage.

Indigenous children and children from other ethnicities with darker complexions will be offered the Ditto™ procedural preparation stories with characters that have darker skin tones. Furthermore, the exposure and frequency of use of other types of video game technologies will be recorded for each participant and correlated with Ditto™ satisfaction.

The caregiver is asked to rate their level of satisfaction with the pain management their child received (from not satisfied to very satisfied) on a visual analogue scale. The caregiver is also given the opportunity to comment on the positive and negative aspects of treatment and this feedback will remain confidential and will not be shown to the clinical treating team. Satisfaction with the Ditto™ during wound care procedures is evaluated by the child rating how much they liked using the Ditto™ on a 10 cm line sliding scale from enjoyment to disinterest, depicted by child-friendly images of a smiley face thumbs up and an unimpressed thumbs down face anchoring each end of the scale.

### Recruitment and withdrawals

#### Sample size

The standard deviation used in sample size calculations was based on a previous study by Miller [33], who found the standard deviation for time to re-epithelialization was four days. Calculations were based on detecting a clinically important difference in time to re-epithelialization between the control and Ditto groups of three days. With a power of 80% and significance level of 0.05, a sample size of 29 participants per group is required.

We expect 10% of eligible participants to dropout before wound re-epithelialization. We anticipate approximately 33% of participants recruited would later be found not to meet the eligibility criteria for the study due to factors including: the need for grafting; use of Entonox™; pre-existing anxiety conditions becoming known; and child protection concerns being raised. Therefore, it is anticipated that approximately 98 participants will need to be recruited in total, in order to achieve final participant numbers of 29 in each group.

#### Randomization

Participants are randomized using a portable computerized random number generator. Randomization is performed by nursing or administration staff members in the Burn Centre who are not associated with the study. The primary researcher is then informed as to which group the participant has been consigned.

### Implementation

Recruitment will take place between 9 August 2011 and 31 August 2012. It is expected the required sample size will be achieved within this time period. Enrolment of participants is carried out by the primary researcher. Children are screened on presentation to the Burn Center for eligibility to this prospective randomized controlled trial. Once it has been established that the child meets all eligibility criteria, the primary researcher approaches the parent/caregiver/s to explain the study and provide them with a copy of the study information sheet. Parent/caregiver/s are encouraged to ask questions. Parents are guided through the informed consent form step-by-step to ensure they understand all aspects of the research project and what participation will involve.

### Blinding

The non-pharmacological intervention received cannot be masked. Assessment of the primary outcome, re-epithelialization, is undertaken by burn wound specialists who are masked to treatment received by the participant.

### Discontinuation/adverse events

Dizziness and nausea are potential adverse effects from engagement in virtual reality. No such effects were reported in previous studies that used the Ditto™ device [33,34,61]. If such effects are experienced by participants, they are free to cease participation if desired. All adverse events will be recorded in both treatment groups.

### Statistical methods

#### Data analysis

All analysis will be conducted using Stata/SE 11 (StataCorp LP, College Station, TX, USA). Analysis will be performed based on the “intention-to-treat” principle, where participants will be analyzed according to the treatment they were allocated. Any dropouts will be excluded from analysis. Participant’s baseline demographic, clinical and social characteristics will be summarized using descriptive statistics. Between-group differences at baseline will be investigated using Fisher’s Exact test (categorical data) or Student’s *t*-test (continuous data). The association between treatment received and healing outcomes will be investigated using regression models. Continuous outcomes will be investigated using linear regression and binary outcomes with logistic regression. If assumptions for linear regression are not met, outcomes will be analyzed using non-parametric tests. Regression models will include treatment group as the only main effect, unless groups are significantly unbalanced at baseline, in which case the regression models will include two main effects (treatment group and time) as well as a treatment-by-time interaction term. The efficacy of the Ditto™ may differ according to age and the number of days to re-epithelialization will be affected by the depth of the burn. Analysis will also be conducted with data stratified for depth of burn (superficial/superficial partial thickness/deep partial thickness/full thickness) and age of participant (for example, under 7 years 11 months/8 years and greater, with age strata based on age group validity of the VAS-A). Where appropriate, repeated-measures analysis will be undertaken using Generalized Estimating Equations. Treatment and time will be included as main effects, and a treatment-by-time interaction will be performed. For continuous outcomes we will assume a Gaussian Family and for binary outcomes the Binomial Family, each with their natural link. An exchangeable correlation structure will be assumed. For all analyses a *P*-value of 0.05 will be considered significant. There will be no adjustment for multiple comparisons.

### Data storage

Data are stored securely by the principal investigator in locked filing cabinets within the secure area of the Queensland Children’s Medical Research Institute, The University of Queensland. Data are entered into an Excel spread sheet. Incomplete data from medical records are checked for and identified when entering data into Excel. All other incomplete data are coded accordingly as missing, unknown or not applicable. The data set will be cleaned and checked before being locked for analysis. On completion of the trial, data will be kept for a period of 15 years in accordance with the ethical requirements of the Queensland Children’s Health Services (RCH) Human Research Ethics Committee.

## Discussion

This trial utilizes a number of measures to investigate the links between the novel Ditto™ procedural preparation and distraction intervention and the patient’s experience of pain, stress and anxiety, to ultimately determine the impact this has on re-epithelialization of acute burn wounds. This is the first RCT in the area of acute burn injuries which examines the relationships among pain, stress, anxiety and re-epithelialization.

The rate of re-epithelialization has discernible implications for the formation of hypertrophic scarring and the long term physical and psychological issues resulting from scarring. The significance of this study is that if the Ditto™ intervention is associated with a reduction in time taken for acute burn wounds to re-epithelialize, patients may heal within the optimal 10 to 14 days, reducing the likelihood of hypertrophic scarring [62].

This trial will also be the first to measure the utility of salivary cortisol and salivary α-amylase as indicators of stress during acute burn wound care procedures. Data collected in the course of this study will seek to answer many questions regarding the pain and stress experienced by burned children, including: do children’s stress and pain levels predict re-epithelialization rate?; are pain and stress levels positively correlated?; and is there an association with age or gender?

There are some limitations with this study, mostly related to dealing with children in pain. There may be challenges with obtaining laser Doppler image (LDI) scans to determine burn wound depth. During scans children are required to remain very still for up to several minutes, ideally with their wounds free of dressings; however, exposing wounds to air flow can cause increased pain for the patient. The Visitrak™ measure involves tracing wounds, which may also be challenging for children not wanting their burns to be touched. The individualized perception of pain poses an additional limitation to measuring Ditto™ effectiveness. Exposure to noxious stimuli as an infant has the potential to permanently change the neuronal architecture of the developing brain, thus resulting in greater pain sensitivity as adolescents [63]. This study will include children with varying experiences and memories of pain, which will shape their experience of and rating of pain levels regardless of Ditto™ engagement. To attempt to diminish this effect, children known to have existing psychological issues, or known to SCAN or child safety are excluded from this study.

The diurnal variation of cortisol may create challenges in the analysis of salivary cortisol. The cortisol awakening peak occurs an hour after waking, and burn clinics occur early in the morning, so when study samples are taken it will be more difficult to identify any peaks in stress levels. Ideally, a within-subjects design is best when comparing highly variable biological markers; however, as procedural preparation is a strong component of this proposed study, a within-subjects design is not feasible.

### Significance of the study

The pain associated with burn injuries and the need for additional treatment to standard pharmacological management is widely known and well established in the literature. Limited staff resources and busy burn clinics are common place, highlighting the need for interventions such as the Ditto™ device, which require very little set-up time. The effectiveness of the Ditto™ device in reducing pain and time taken for burn wound care procedures is well established [33,34]. The potential of this device to also improve wound healing is of great significance in the burns field, possibly leading to decreased risk of scarring and scar management requirements and perhaps the difference between grafting and not grafting more severe burns. Establishing a link between reduced pain, stress and anxiety and improving healing time in acute burn wounds would be of major significance for patients and health care providers, and has application for all health care procedures which require pain/stress management, not just acute burns.

## Trial status

This trial is currently continuing to recruit participants and collect data. The cessation of participant recruitment is planned for 31 August 2012 and data collection is likely to continue to January 2013 (with data collection continuing until three months post re-epithelialization of participant’s burns).

## Abbreviations

COD: Change of dressings; CTSQ: Child Trauma Screening Questionnaire; DTT: Diversionary Therapy Technologies; FLACC: Face, Legs, Arms, Cry, Consolability; FPS-R: Faces Pain Scale – Revised; HR: Heart rate; INOS: Inducible nitric oxide synthase; LDI: Laser Doppler image; MMPs: Matrix metalloprotinases; PTSD: Post-traumatic stress disorder; QCMRI: Queensland Children’s Medical Research Institute; RCH: Royal Children’s Hospital; SCAN: Suspected child abuse and neglect; TBSA: Total body surface area; VAS-A: Visual Analog Scale–Anxiety; VR: Virtual reality.

## Competing interests

This clinical trial was partially financially supported by a grant given to the Royal Children’s Hospital, Brisbane, by Diversionary Therapy Technologies (DTT). Despite this financial support, DTT had no part in the study design and data collection of this project, nor will they have any involvement in the analysis or publication of results. One of the supervisors of the trial, Roy Kimble, holds options with DTT; however, he will not stand to lose or gain financially or personally from the results during the clinical trial period and time of submission. The principal researcher has no financial interest in the Ditto™ device or the DTT company and remains an employee of the Royal Children’s Hospital, Brisbane.

## Authors’ contributions

NJB, LC, RMK and SR all made substantial contributions to the design of this trial. RSW has made substantial contributions to the statistical design and wrote the data analysis in this manuscript. NJB wrote the draft manuscript with substantial input from LC. All authors provided critical review of the article and approved the final manuscript.
